# A hybrid machine learning approach for detecting DDoS attacks in software-defined networks

**DOI:** 10.1038/s41598-026-35458-w

**Published:** 2026-01-28

**Authors:** Iftekhar Ahmed Mahar, Kamran Aziz, Prasun Chakrabarti, Naveed Ahmed, Mohamad Ladan, Yasir Javed

**Affiliations:** 1 School of Computer Science, Wuhan University, Wuhan, 430000 China; 2 Digital Technologies, Hainan Bielefeld University of Applied Sciences, Danzhou, Hainan 578001 China; 3 Department of Computer Science and Engineering, Sir Padampat Singhania University, Udaipur, 313601 Rajasthan India; 4 College of Computer and Information Sciences, Prince Sultan University, Riyadh, 8923 Saudi Arabia

**Keywords:** Software-defined networking (SDN), Distributed denial of service (DDoS), OpenFlow, Flow statistics, Port statistics, Feature engineering, Machine learning, Hybrid classification model, Random forest, XGBoost, Traffic classification, SDN security, Engineering, Mathematics and computing

## Abstract

Software-Defined Networking (SDN) introduces programmability and centralized control to modern networks, but this flexibility also exposes both the controller and data plane to severe threats such as Distributed Denial of Service (DDoS) attacks. Effective early detection of these attacks requires SDN-aware traffic features that capture the unique behavior of OpenFlow-based environments. This study presents a machine-learning framework for distinguishing benign and malicious traffic using a dataset constructed directly from an SDN testbed employing a Ryu controller and Open vSwitch. Flow and port-level statistics were periodically collected through OpenFlow monitoring messages, enabling the extraction of new SDN-specific features tailored for DDoS detection. A hybrid classification model that integrates the Random Forest (RF) with XGBoost (XGB) Classifier is proposed to enhance detection performance. The hybrid RF-XGB model demonstrates clear superiority over individual classifiers, achieving an accuracy of 99.36% and exhibiting near-perfect discrimination in ROC AUC and confusion matrix evaluations. These results confirm that combining SDN based feature engineering with ensemble learning provides a highly effective and reliable approach for early DDoS detection in programmable networks.

## Introduction

**Fig. 1:**
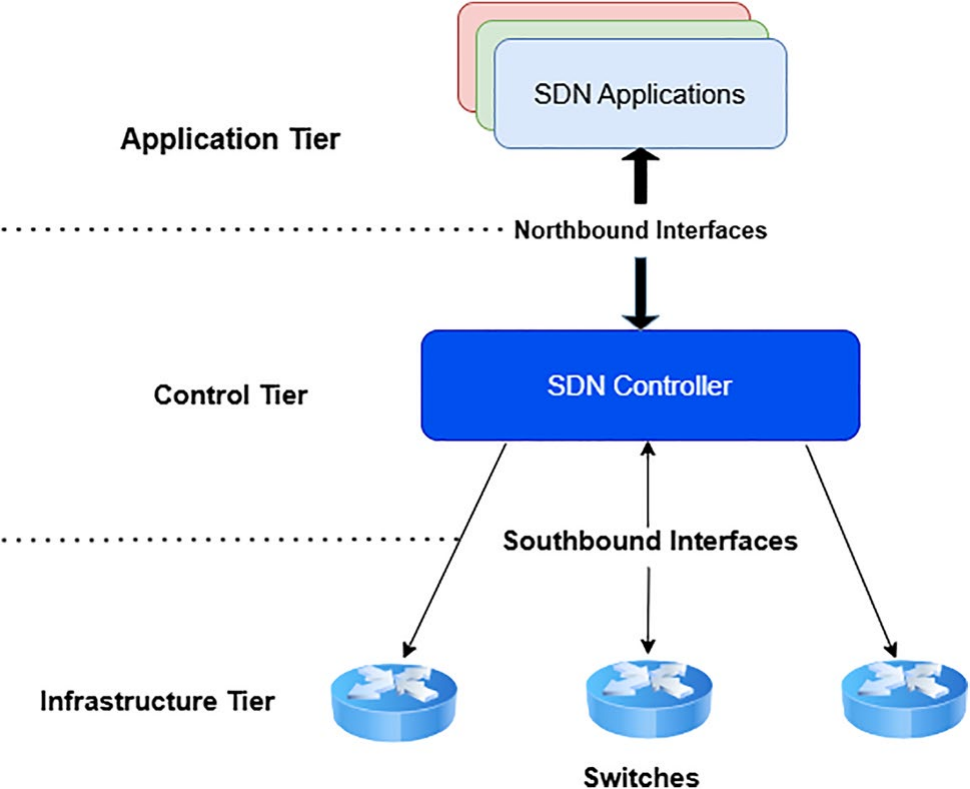
Software defined network (SDN) typical architecture.

Software-Defined Networking (SDN) has emerged as a transformative paradigm in modern communication networks by decoupling the control plane from the data plane, enabling centralized control, programmability, and dynamic configuration^1,2^ (see Fig. 1). Unlike traditional networks where routers and switches handle both forwarding and control functions, SDN leverages controllers such as Floodlight, Ryu, Pox, and OpenDaylight to manage network policies centrally^3–6^. This architecture reduces complexity, lowers costs by supporting vendor independence, and enhances agility for applications in data centers, cloud systems, IoT, and 5G networks^7,8^.Recent studies have also emphasized that SDN’s programmability increases both its efficiency and its exposure to sophisticated network attacks, particularly when adversaries exploit controller-centric vulnerabilities^9^. However, while SDN improves efficiency, its centralized design and reliance on open APIs also introduce unique vulnerabilities across multiple layers^10,11^.

Recent advancements in artificial intelligence have demonstrated strong potential for enhancing cybersecurity incident response by enabling adaptive threat detection and automated decision-making. For instance, Ali et al.^12^ proposed an AI-driven optimization framework that significantly improves the detection of advanced persistent threats by dynamically analyzing large-scale network behavior, highlighting the effectiveness of intelligent learning mechanisms against evolving cyber threats.

In parallel, Distributed Denial of Service (DDoS) attacks have evolved into one of the most disruptive cyber threats. Unlike traditional DoS attacks launched from a single machine, DDoS attacks harness botnets of compromised devices to overwhelm targets with malicious traffic^13–15^. These attacks cause downtime, financial losses, and reputational damage while also serving as diversions for more stealthy intrusions such as data theft^16–19^. In the SDN context, the controller itself becomes a high-value target, making the entire network vulnerable to a single point of failure^20,21^. By exploiting SDN’s centralized architecture and open interfaces, attackers can disrupt critical services at scale, raising the urgency of robust defense mechanisms^22–24^. Furthermore, empirical evaluations show that DDoS campaigns targeting SDN controllers can escalate rapidly due to the architectural coupling of event processing and flow-rule installation, making early detection essential for preserving network availability^25^. Recent advances in artificial intelligence have further highlighted the role of adaptive and automated response mechanisms in strengthening cyber defense infrastructures. For instance, AI-driven optimization frameworks have been shown to significantly enhance incident response efficiency and threat detection accuracy when dealing with persistent and stealthy attack behaviors^12^. Such findings reinforce the need for intelligent and adaptive detection strategies within SDN environments, particularly against evolving DDoS threats.

Several recent works have demonstrated that combining statistical profiling with machine learning significantly improves the detection of high-rate and low-rate DDoS traffic in SDN environments^26^. Extensive efforts from academia and industry have focused on addressing this challenge, with approaches ranging from traditional filtering to advanced intrusion detection systems. Recently, machine learning (ML) has shown promise in detecting anomalous traffic patterns by learning from large-scale SDN traffic data^27–29^. However, existing ML-based models often suffer from scalability issues, high false positive rates, and limited adaptability to new or stealthier DDoS variants^30,31^. Moreover, many proposed solutions rely on outdated datasets such as NSL-KDD that lack SDN-specific features, reducing their real-world applicability^32^. These limitations highlight the need for hybrid approaches that combine the strengths of multiple algorithms while addressing dataset and adaptability constraints.Hybrid detection models have been shown to outperform single-classifier approaches by leveraging complementary feature learning capabilities, especially under diverse SDN workloads^33^.

To address these limitations, this study introduces a hybrid machine learning framework for the accurate detection and prevention of DDoS attacks in SDN environments. The key contributions of this work are as follows: (i) the development of a lightweight and effective hybrid architecture that integrates optimized Random Forest and XGBoost classifiers for enhanced detection performance; (ii) the construction and evaluation of an SDN-specific dataset derived from real-time OpenFlow statistics, incorporating engineered features tailored to SDN traffic behavior; and (iii) a comprehensive assessment demonstrating improved scalability, reduced false positives, and strong resilience against diverse attack patterns when compared with existing approaches.

Experimental results show that the proposed hybrid model achieves an accuracy of 99.36%, with excellent precision, recall, and generalization capability, establishing it as a practical and high-performance solution for securing modern SDN deployments.

## Literature review

### Early and traditional machine learning approaches

Initial attempts to detect DDoS attacks in SDN leveraged statistical thresholds and flow-based monitoring. Buragohain et al.^34^ proposed restricting user requests by recording maximum and minimum flow durations and traffic rates, flagging anomalies as malicious. Similarly, Da Silva et al.^35^ detected attacks by monitoring sudden increases in flow counts and memory usage, achieving modest accuracy 83%. While lightweight, these methods are limited by static thresholds and fail to adapt to dynamic traffic variations. Similar findings were reported in^36^, where threshold-based detection approaches were shown to degrade significantly under fluctuating SDN traffic loads.

**Fig. 2:**
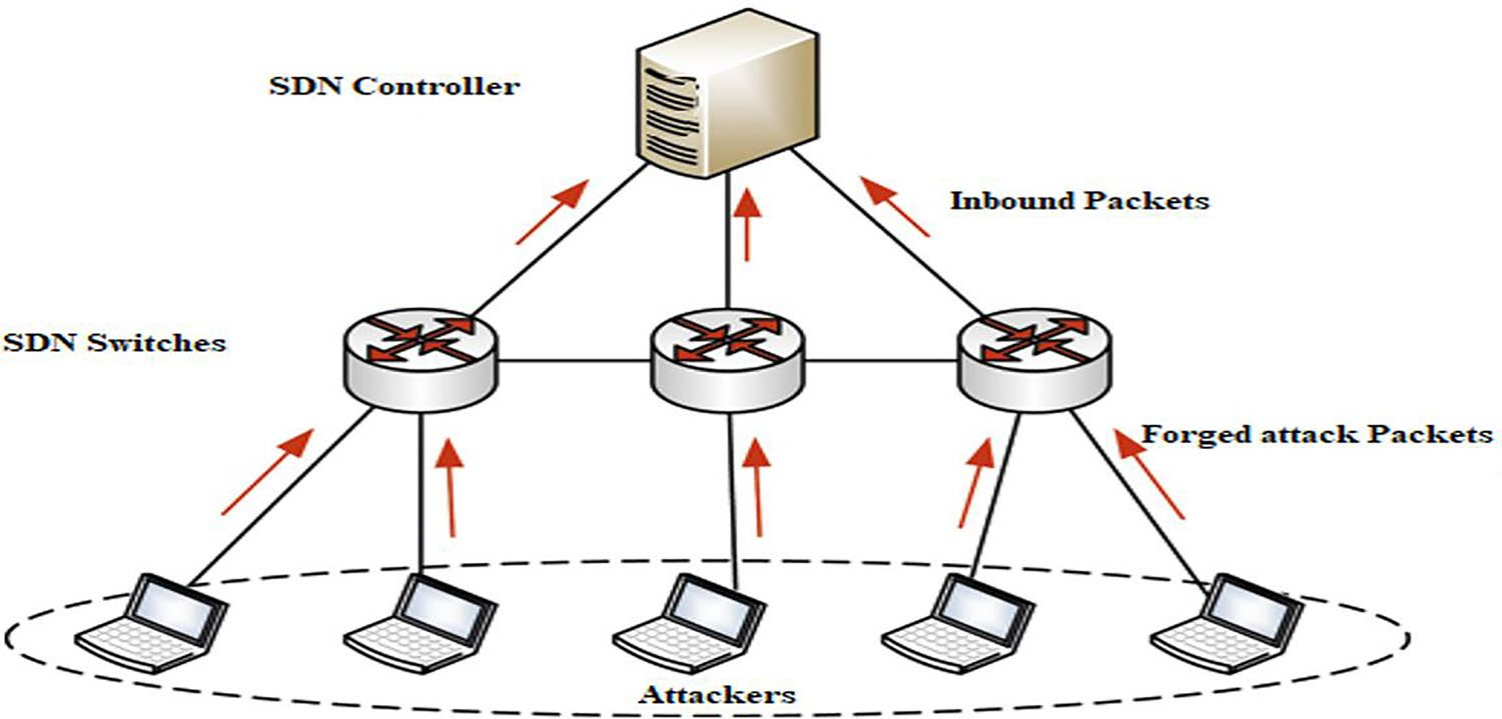
DDoS attack on a Software defined network (SDN).

The integration of ML marked a shift toward data-driven approaches. Latah and Toker^32^ evaluated Decision Trees, Random Forests, Naïve Bayes, KNN, and Neural Networks on the NSL-KDD dataset. Neural Networks provided the best accuracy, but reliance on legacy datasets with non-SDN-specific features limited applicability. Rasool et al.^37^ focused on link flooding detection in SDN using Multi-Layer Perceptrons (MLP) and OpenFlow statistics, demonstrating better performance but still constrained by limited feature diversity. Such DDoS attack scenarios in SDN, where botnets flood the centralized controller or switches, are illustrated in Figure 2.

### Advanced learning techniques

To address dimensionality, Chaturvedi et al.^38^ introduced a tensor-based framework leveraging eigentensors and decomposition techniques. This enabled detection of anomalies across higher-order correlations such as source/destination IPs, ports, and packet counts. While tensor methods improve representation, they demand heavy computation and may be impractical for real-time SDN environments.

Hybrid ML approaches have gained traction for balancing accuracy and efficiency. Rani et al.^29^ demonstrated that deep learning models outperform classical classifiers in identifying complex attack patterns, though at the expense of higher computational cost. More recently, adaptive self-learning mechanisms deployed at the network edge have been explored to mitigate DDoS attacks under resource-constrained environments. Hussain et al.^39^ proposed an Edge AI-based self-learning framework capable of dynamically adapting to evolving attack patterns, achieving improved detection accuracy and reduced latency in wireless and distributed network settings. More recently, Rezaei et al. (2025) emphasized the role of hybrid feature selection and ensemble models like Random Forest in improving detection under imbalanced data. Similarly, Abiramasundari and Ramaswamy (2025) combined PCA with classifiers including RF and KNN on CICIDS datasets, reporting 98.9% accuracy, showing that carefully engineered classical ML can match deep learning. Sawah et al. (2025) compared multiple classifiers with feature elimination and again found ensemble models consistently superior. In addition, recent computational studies have validated that ensemble-based hybrid detectors can maintain high predictive stability even when facing imbalanced SDN traffic distributions^40^.

### Deep learning frameworks and research challenges

Deep architectures have also been applied to capture temporal and spatial features in traffic. Elshewey et al. (2025) proposed a hybrid CNN–GRU model with preprocessing techniques such as SMOTE to address class imbalance, achieving state-of-the-art accuracy. Meliboev (2025) explored CNN, LSTM, and GRU combinations on CICDDoS2019, highlighting the importance of generalization to unseen attacks. Karrakchou et al. (2025) introduced an innovative early-exit neural network within P4-SDN, running quantized CNNs in the data plane and GRUs in the control plane, reducing inference latency and controller load. Recent ensemble-based deep learning models have further improved intrusion detection performance by combining temporal and policy-driven learning. Hussain et al.^41^ introduced an ensemble framework integrating deep reinforcement learning and LSTM networks, demonstrating superior adaptability and robustness against both known and unseen attack behaviors. These findings support the effectiveness of hybrid and ensemble learning paradigms in addressing the dynamic nature of DDoS attacks. Federated and privacy-preserving approaches have also emerged, e.g., GAN-based cross-domain detection for heterogeneous SDNs, though these remain in early stages.

Despite high reported accuracies (often above $98\%$), limitations persist. Many models rely on outdated or simulation-only datasets, raising concerns about real-world deployment. Others focus on detection without addressing mitigation latency or prevention strategies. Deep models, while powerful, are computationally expensive and less interpretable, limiting trust in critical infrastructures. To mitigate these limitations, some researchers have begun incorporating reinforcement-learning-assisted mechanisms and adaptive decision policies, which enhance resilience against evolving DDoS behaviors^42^. Moreover, scalability across large-scale SDNs and adaptability to evolving attack vectors remain unresolved challenges.

### Existing datasets

This study reviews publicly available DDoS attack datasets spanning 2000 to 2024, emphasizing the urgent requirement for reliable and comprehensive resources to support the evaluation of detection systems. While numerous intrusion detection datasets exist, many are synthetically produced or simulation-based, leading to unrealistic traffic characteristics and incomplete protocol coverage. The earliest benchmark in this domain was the DARPA dataset, released by Lincoln Laboratory in 1998^43^. Over the years, several others followed, including DEFCON (2000–2002), KDD99 (1998–1999), NSL-KDD (2009), CAIDA (2000–2016), and Kyoto (2009). Each of these, however, suffers from drawbacks such as limited traffic variety, insufficient data volume, or simplified attack environments. To address these shortcomings, Sharafaldin et al. highlighted the deficiencies of prior datasets^43^ and introduced CICIDS2017, which remains among the most complete resources for intrusion detection research. Building on this foundation, the DDoS2019 dataset^13^ was developed using a full network topology to generate diverse attack scenarios. The traffic was processed through CICFlowMeter, a tool that extracts more than 80 bidirectional flow features, such as packet counts, byte totals, lengths, and timing statistics. The background traffic in CICIDS2017 was simulated across 25 users interacting via FTP, SSH, HTTP, HTTPS, and email protocols, while DDoS instances were produced using LOIC. A notable limitation of this dataset is the absence of genuine raw network captures. More recently, the lack of domain-specific and realistic datasets has been addressed through targeted dataset development efforts. Hadi et al.^44^ introduced UAV-NIDD, a dynamic intrusion detection dataset designed for UAV networks, emphasizing realistic traffic modeling and diverse attack scenarios.

Creating truly representative datasets requires environments that replicate dynamic client behaviors with both legitimate and malicious traffic, encompassing thousands of real attack traces and applications in real time. In line with this requirement, Hadi et al.^45^ developed a realistic distributed denial-of-service dataset for machine learning-based intrusion detection systems, capturing diverse DDoS attack types and traffic intensities to improve model generalization and real-world applicability. In line with this, Iman et al. constructed a more realistic DDoS benchmark^13^ by employing modern simulators such as HOIC, Slowloris, DDoSIM, HULK, Goldeneye, Bonesi, Mirai Botnet, and Tor Hammers, thereby reducing reliance on complete physical infrastructures.

## Methods and data source

### Data source

The dataset used in this study was generated directly from an OpenFlow-enabled SDN environment by periodically collecting both flow-level and port-level statistics from the switches. The Ryu controller issued OFPFlowStatsRequest and OFPPortStatsRequest messages at fixed intervals, enabling the extraction of attributes such as flow duration, packet counters, flow-table occupancy, protocol type, and per-port throughput measurements. Each entry in the dataset corresponds to a single flow observation captured during these polling intervals.

All flow records were automatically annotated within the controller: flows observed under normal network operation were labeled as benign (“0”), whereas flows captured during the execution of DDoS attack scripts were labeled as malicious (“1”). This automated labeling mechanism eliminates manual subjectivity and ensures consistency throughout the dataset.

The final dataset contains **99,225 rows** and **22 SDN-specific attributes** which is considered a medium-sized dataset for SDN-based DDoS detection research. These attributes collectively capture traffic behavior under both benign and attack conditions, forming the basis for the machine learning analysis conducted in this study.

### Testbed and dataset generation

A software-defined networking (SDN) test environment was implemented using Mininet, consisting of a single Open vSwitch (OVS) connected to multiple virtual hosts and managed by a centralized Ryu controller. The simplified single-switch topology was intentionally selected to ensure reproducibility and precise control over network behavior, while still supporting fundamental OpenFlow operations such as flow monitoring and statistics exchange.

For each experiment, the controller periodically gathered flow-level and port-level statistics from the switch. The recorded dataset includes temporal, traffic, and protocol-related features such as:

- flow duration measured in seconds and nanoseconds (dur, dur_nsec, tot_dur),
- source and destination identifiers (src, dst),
- protocol type (Protocol),
- packet and byte counters (pktcount, bytecount),
- packet arrival rate (pktrate),
- transmitted and received byte volumes (tx_bytes, rx_bytes),
- per-port throughput statistics in kilobits per second (tx_kbps, rx_kbps, tot_kbps),
- number of active flows observed at the switch (flows),
- packet-in events generated by the switch (packetins).

Normal network behavior was generated using standard ICMP, TCP, and UDP communications between hosts. In contrast, attack scenarios were emulated by launching high-rate traffic patterns, including UDP floods, ICMP floods, and TCP-based flooding attacks, aimed at stressing the control and data planes. Each flow instance was labeled to distinguish benign and malicious traffic, and the resulting measurements were stored in structured CSV format for subsequent analysis.

### Experimental scenarios

Three SDN-relevant DDoS attack scenarios were executed in the testbed: protocol-based flooding attacks (TCP SYN/ACK floods), volumetric flooding attacks (UDP and ICMP floods), and mixed flooding attacks combining multiple traffic types. These scenarios were selected because of their known impact on SDN infrastructures, particularly their ability to overload switches and controllers by generating excessive flow arrivals and packet-in events.

Benign traffic was generated concurrently to emulate realistic network behavior. The coexistence of benign and malicious traffic introduced natural variations in flow counts, port utilization, and *estimated bandwidth utilization*, thereby increasing dataset diversity and improving the generalization capability of the trained machine learning models.

### Testing methodology

Each experiment involved generating traffic while the Ryu controller recorded flow-level and port-level statistics. All collected attributes were directly stored in CSV format, and each flow entry was automatically assigned a binary label. Multiple experimental runs were conducted to introduce temporal variation and avoid overfitting to a single traffic pattern.

This methodology produced a dataset that accurately reflects real SDN behaviour under both normal and DDoS conditions, enabling robust evaluation of machine learning models.

### Feature extraction and engineering

The constructed dataset comprises **22 features** extracted from OpenFlow statistics and augmented with SDN-specific engineered attributes. These features are grouped into three categories:

- **Flow-Level Features:** Flow duration metrics (dur, dur_nsec, tot_dur), packet and byte counters (pktcount, bytecount), and the number of active flows (flows), which capture traffic amplification behavior.
- **Switch and Protocol Features:** Protocol type (Protocol), packet-in events (packetins), and port- and flow-related identifiers that reflect controller–data plane interactions.
- **Rate and Estimated Bandwidth Features:** Per-port throughput statistics (tx_kbps, rx_kbps, tot_kbps), transmitted and received byte volumes (tx_bytes, rx_bytes), and derived metrics such as packet arrival rate and *estimated bandwidth utilization*, which are indicative of high-rate attack traffic.

Two aggregate SDN features are further computed: the *Average Packet Count per Flow (APPF)* and the *Average Byte Count per Flow (ABPF)*, given by

1 $$\begin{aligned} APPF = \frac{\texttt {pktcount}}{\texttt {flows}}, \qquad ABPF = \frac{\texttt {bytecount}}{\texttt {flows}}. \end{aligned}$$

The packet arrival rate is derived from consecutive packet observations as

2 $$\begin{aligned} PacketRate = \frac{p_{t+1} - p_t}{\Delta t}, \end{aligned}$$

while *estimated bandwidth utilization* is computed from transmitted byte counts as

3 $$\begin{aligned} EstimatedBandwidth = \frac{\texttt {tx\_bytes} \times 8}{1000}. \end{aligned}$$

Together, these features provide a concise yet comprehensive representation of SDN traffic behavior, enabling effective discrimination between benign and DDoS attack traffic.

### Proposed method

**Fig. 3:**
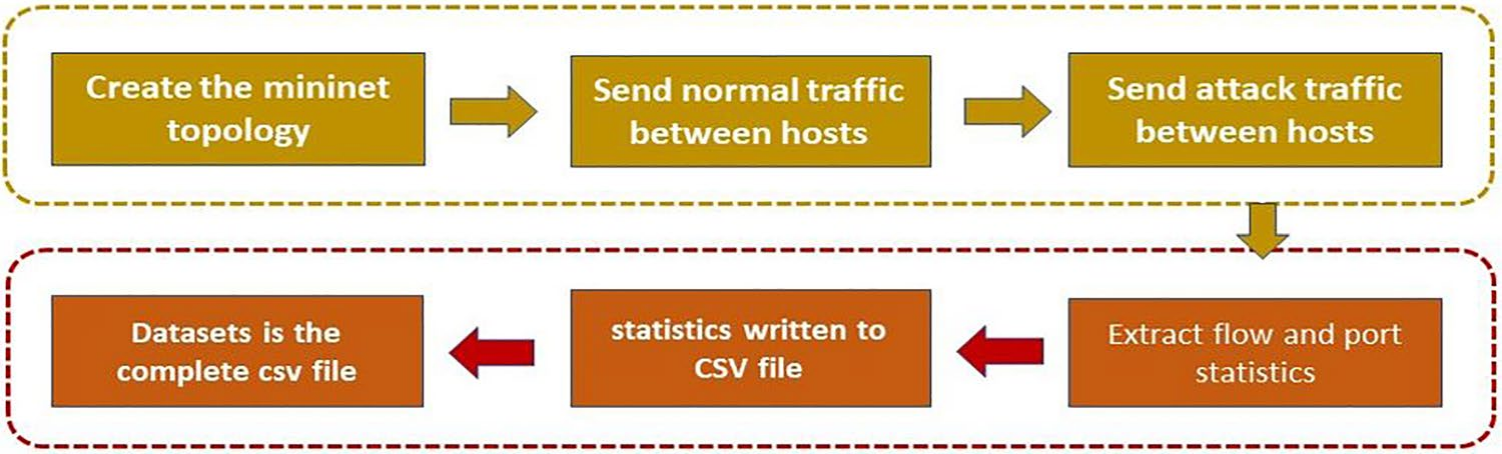
Dataset construction framework model.

This subsection outlines the procedure used to construct the SDN traffic dataset and prepare it for machine learning analysis. The proposed method is based on an OpenFlow-driven monitoring framework in which a Ryu controller periodically collects traffic statistics from an Open vSwitch (OVS). The overall dataset generation workflow is summarized in Algorithm 1 and illustrated in Fig. 3.

**Algorithm 1:**
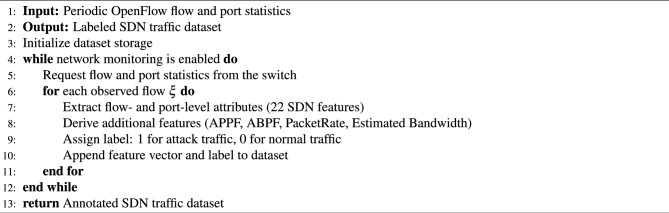
SDN traffic dataset construction.

During operation, the controller periodically issues OFPFlowStatsRequest and OFPPortStatsRequest messages to collect flow- and port-level statistics from the OpenFlow switch. Flow-level data include duration metrics, packet and byte counters, and the number of active flows, while port-level measurements capture transmitted, received, and aggregate throughput (tx_kbps, rx_kbps, tot_kbps) and raw byte counts (tx_bytes, rx_bytes) to characterize traffic intensity and *estimated bandwidth utilization*.

All extracted statistics are recorded at fixed sampling intervals and stored in structured CSV files. Traffic labels are automatically assigned by the controller based on the active traffic scenario, ensuring consistency, reproducibility, and a clear distinction between benign and malicious traffic.

Figure 3 illustrates the complete dataset construction pipeline, including controller–switch communication, statistics extraction, storage, and labeling. This workflow ensures that the collected data accurately reflects SDN behavior under both normal and DDoS attack conditions.

**Fig. 4:**
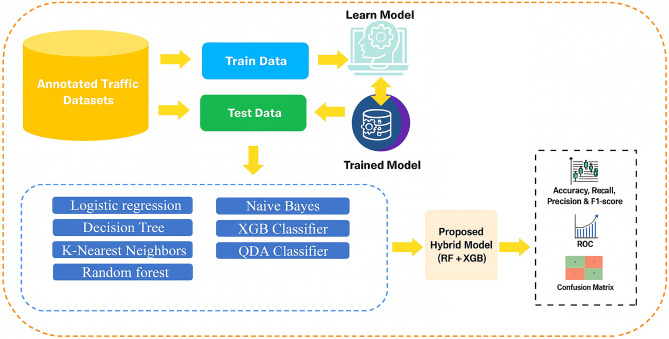
Classification and preprocessing workflow.

Once data collection is complete, preprocessing steps are applied before model training. Figure 4 provides an overview of the data preparation workflow. Preprocessing includes handling missing values, removing incomplete rows, encoding the protocol field numerically, and normalizing numerical attributes to ensure uniform contribution during training.

The final dataset consists of **99,225 rows and 22 attributes**. After preprocessing, an 80:20 train–test split was used. Exploratory analyses, including correlation heatmaps and descriptive statistics, were conducted to understand relationships among attributes and assess their discriminative potential for machine learning-based DDoS detection.

## Experimental design work

All experiments were conducted on a Windows-based HP laptop equipped with a 64-bit processor and 16 GB of RAM. The SDN simulation environment consisted of a single Ryu controller connected to an Open vSwitch (OVS) instance, with multiple Mininet hosts attached to the switch. This lightweight yet flexible setup enabled controlled generation and observation of both benign and malicious traffic flows.

Benign traffic was produced using the mgen tool at a rate of approximately 450 packets per second, with flows originating from randomly selected hosts to emulate diverse communication patterns. Malicious traffic was generated using the hping3 utility^46^, also configured at 450 packets per second, to maintain consistency between benign and attack conditions. The attacker employed spoofed source IPs to increase flow-table pressure and emulate realistic DDoS-style behavior.

During each monitoring interval, the Ryu controller issued OFPFlowStatsRequest and OFPPortStatsRequest messages to the switch to retrieve updated OpenFlow statistics. A dedicated reply handler processed these responses and stored the extracted values into a comma-separated values (CSV) dataset. The recorded attributes included raw flow-level metrics, port-level bandwidth indicators, and several derived traffic features used later for machine learning (see Table 1).

**Table 1:** Attributes extracted from the SDN dataset.

No.	Attribute	No.	Attribute
1	Timestamp (dt)	12	Packet Count (pktcount)
2	Switch Identifier (switch)	13	Byte Count (bytecount)
3	Flow Duration (sec) (dur)	14	Avg. Packets per Flow (APPF)
4	Flow Duration (nsec) (dur_nsec)	15	Avg. Bytes per Flow (ABPF)
5	Active Flow Count (flows)	16	Packet Rate (pktrate)
6	Protocol Type (Protocol)	17	TX Throughput (tx_kbps)
7	Port Number (port_no)	18	RX Throughput (rx_kbps)
8	Source IP (src)	19	Total Bandwidth (tot_kbps)
9	Destination IP (dst)	20	TX Byte Counter (tx_bytes)
10	Source MAC (src_mac)	21	RX Byte Counter (rx_bytes)
11	Destination MAC (dst_mac)	22	Class Label (label)

The monitoring interval was set to 30 seconds for all experiments, although this parameter remains dynamically configurable due to SDN programmability. The retrieved OpenFlow statistics revealed clear distinctions between benign and malicious traffic, with attack flows exhibiting higher packet rates, increased numbers of active flows and packet-in events, and elevated estimated bandwidth utilization.

The complete dataset consists of 99,225 rows and 22 attributes. Benign and malicious traffic were generated across 10 independent network runs, each lasting approximately 1500 seconds, resulting in over 250 minutes of experimental activity. Benign traffic included UDP datagrams, TCP connections, and ICMP echo messages, while DDoS attack scenarios consisted of UDP floods, TCP SYN floods, and ICMP flooding patterns. All collected flow- and port-level statistics were stored directly in CSV format.

## Performance parameters

To evaluate the effectiveness of the proposed hybrid model, the constructed SDN dataset was used to train and validate multiple machine learning classifiers. Their performance was assessed using widely adopted evaluation metrics, including Accuracy, Precision, Sensitivity (Recall), Specificity, F1-score, and the Matthews Correlation Coefficient (MCC). These metrics are derived from the confusion matrix shown in Table 2, which summarizes the relationship between predicted and actual class labels.

In a binary classification setting such as benign versus malicious flow detection, the confusion matrix contains four primary components:

- **True Positive (TP):** Malicious traffic correctly identified as malicious.
- **True Negative (TN):** Benign traffic correctly classified as benign.
- **False Positive (FP):** Benign traffic incorrectly classified as malicious (false alarm).
- **False Negative (FN):** Malicious traffic incorrectly classified as benign (missed detection).

These four values form the basis of all performance metrics used in this study. Accuracy measures the proportion of correctly classified samples, while Sensitivity (Recall) reflects the model’s ability to detect malicious flows. Specificity quantifies how effectively the classifier identifies benign traffic. MCC provides a balanced evaluation of classifier performance, making it particularly suitable for datasets with class imbalance.

The evaluation metrics are mathematically defined as follows:

4 $$\begin{aligned} \text {Sensitivity} = \frac{TP}{TP + FN}, \end{aligned}$$

5 $$\begin{aligned} \text {Specificity} = \frac{TN}{TN + FP}, \end{aligned}$$

6 $$\begin{aligned} \text {Accuracy} = \frac{TP + TN}{TP + FP + TN + FN}, \end{aligned}$$

7 $$\begin{aligned} \text {MCC} = \frac{TP \cdot TN - FP \cdot FN}{\sqrt{(TP + FP)(TP + FN)(TN + FP)(TN + FN)}}. \end{aligned}$$

For SDN-based intrusion detection systems, high Sensitivity is critical because it ensures rapid and reliable detection of DDoS activity. Likewise, high Specificity prevents unnecessary disruptions by avoiding false alarms on legitimate flows. Together, these metrics provide a comprehensive assessment of classifier performance in real-time programmable networks.

**Table 2:** Confusion matrix structure.

Actual Malicious (Positive)	Actual Benign (Negative)
True Positive (TP)	False Positive (FP)
False Negative (FN)	True Negative (TN)

Experimental results demonstrate that the proposed hybrid Random Forest–XGBoost model consistently outperforms all baseline classifiers across each metric. Its superior detection capability highlights its suitability for SDN environments, where accurate and timely flow characterization is essential for mitigating the impact of DDoS attacks.

## Results

This section summarizes the performance of the proposed hybrid model using the SDN dataset described earlier. The distribution of benign and malicious traffic is presented in Table 3, followed by the breakdown of traffic classes and their corresponding labels in Table 4. These descriptive statistics provide an overview of dataset composition and support the subsequent evaluation of classification models.

**Table 3:** Number of Instances in the Dataset.

Traffic Class	Number of Instances
ICMP Traffic	39,256
TCP Traffic	27,588
UDP Traffic	32,381
Benign	59,418
Malicious	39,807
Total	99,225

These statistics reveal a balanced representation across protocol types, with a reasonable variation between benign and attack flows. This diversity contributed to stable classifier performance and minimized the risk of bias toward any single traffic category.

**Table 4:** Traffic category of each traffic instance.

Traffic Class	Benign	Malicious
ICMP	23,395	15,861
TCP	16,582	11,006
UDP	19,441	12,940
Total	59,418	39,807

Machine learning models were trained using the dataset and evaluated using Accuracy, Precision, Recall, and F1-score. Simpler models such as Logistic Regression (LR) and Naïve Bayes (NB) showed lower accuracy due to their limited ability to capture nonlinear relationships in flow-level features. In contrast, tree-based models such as Random Forest (RF) and XGBoost (XGB) performed significantly better, benefiting from their capability to model complex interactions between statistical attributes.

The performance of the evaluated classifiers is summarized in Table 5. The proposed hyper-tuned hybrid RF–XGB approach achieved the highest classification accuracy of 99.36%. RF delivered consistently high recall and precision, while XGB provided strong robustness against misclassification errors. These findings demonstrate that ensemble-based learners are particularly effective for SDN DDoS detection.

**Table 5:** Performance measures of different algorithms.

Algorithm	Accuracy	Precision	Recall	F1-Score
Logistic Regression	71.70%	0.71	0.62	0.65
Decision Tree	83.79%	0.76	0.63	0.77
K-Nearest Neighbors	89.88%	0.90	0.86	0.88
Random Forest	98.24%	0.98	0.98	0.98
Naive Bayes	98.00%	0.47	0.99	0.64
XGB Classifier	98.00%	0.99	0.97	0.98
QDA Classifier	59.00%	0.70	0.20	0.31
**Hybrid RF + XGB**	**99.36%**	**0.99**	**0.99**	**0.99**

### Comparative analysis with existing results

The proposed model was compared with prior studies that evaluated SDN-based DDoS detection using emulated datasets. Table 6 summarizes the reported accuracy values from existing literature. As shown, the proposed hybrid model exceeds the performance of previously published techniques, achieving an accuracy of 99.36%.

**Table 6:** Comparison results of traffic classification using various simulated SDN datasets.

S.No	Authors	Year	Accuracy
1	Meit^47^		80%
2	Da Silva^48^		88.7%
3	Perez-Diaz^49^		95%
4	Ye^50^		95.24%
5	Hadem^51^		95.9%
6	Ko^52^		96%
7	Han^53^		96%
8	Myint^54^		97%
9	Ahuja^55^		98.8%
10	Logeswari^56^		98.7%
11	Mahzar^57^		97–99%
12	**Proposed**		**99.36%**

### Observation and discussion

The experimental results demonstrate that the proposed hybrid model accurately differentiates benign and malicious SDN flows by leveraging flow- and port-level OpenFlow statistics along with engineered features such as APPF and ABPF. As shown in Table 5, both RF and XGB demonstrate strong predictive capabilities, and their hybrid integration yields the highest overall performance.

Analysis of estimated bandwidth utilization indicated that during attack periods, aggregate throughput increased substantially, which is consistent with DDoS traffic behavior. These observations support the effectiveness of the proposed model in identifying high-rate attack traffic in SDN environments.

### Receiver operating characteristic (ROC) and AUC performance

**Fig. 5:**
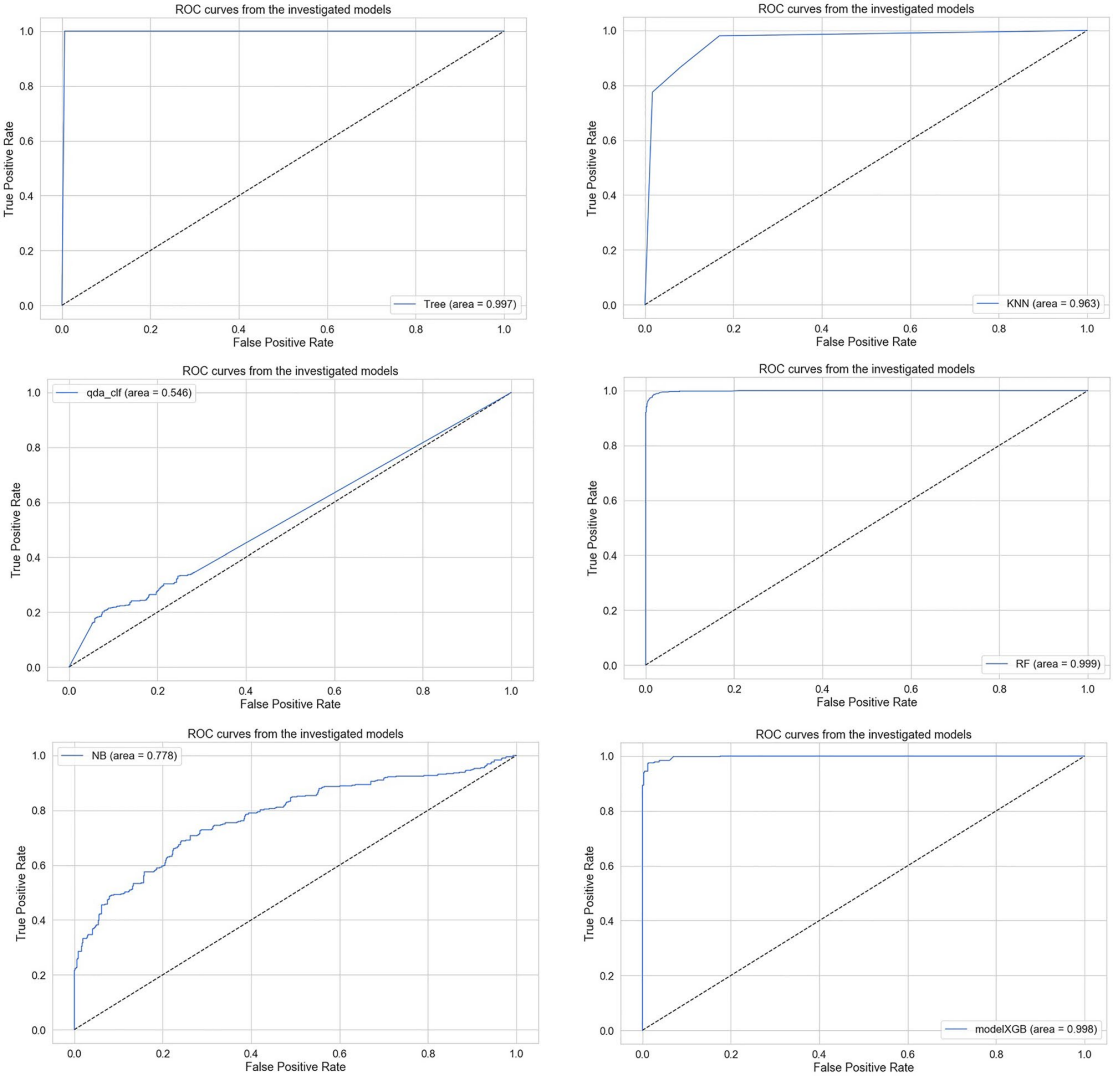
ROC(AUC) performance of different classification models.

The performance of the different models was further analyzed using Receiver Operating Characteristic (ROC) curves and the Area Under the Curve (AUC). ROC curves illustrate the trade-off between True Positive Rate (TPR) and False Positive Rate (FPR) across different classification thresholds, providing a robust measure of discriminative capability. As shown in Fig. 5, models such as RF, XGB, and KNN achieve high AUC values, indicating strong detection performance even under varying thresholds.

### Accuracy assessment using fivefold cross-validation

**Fig. 6:**
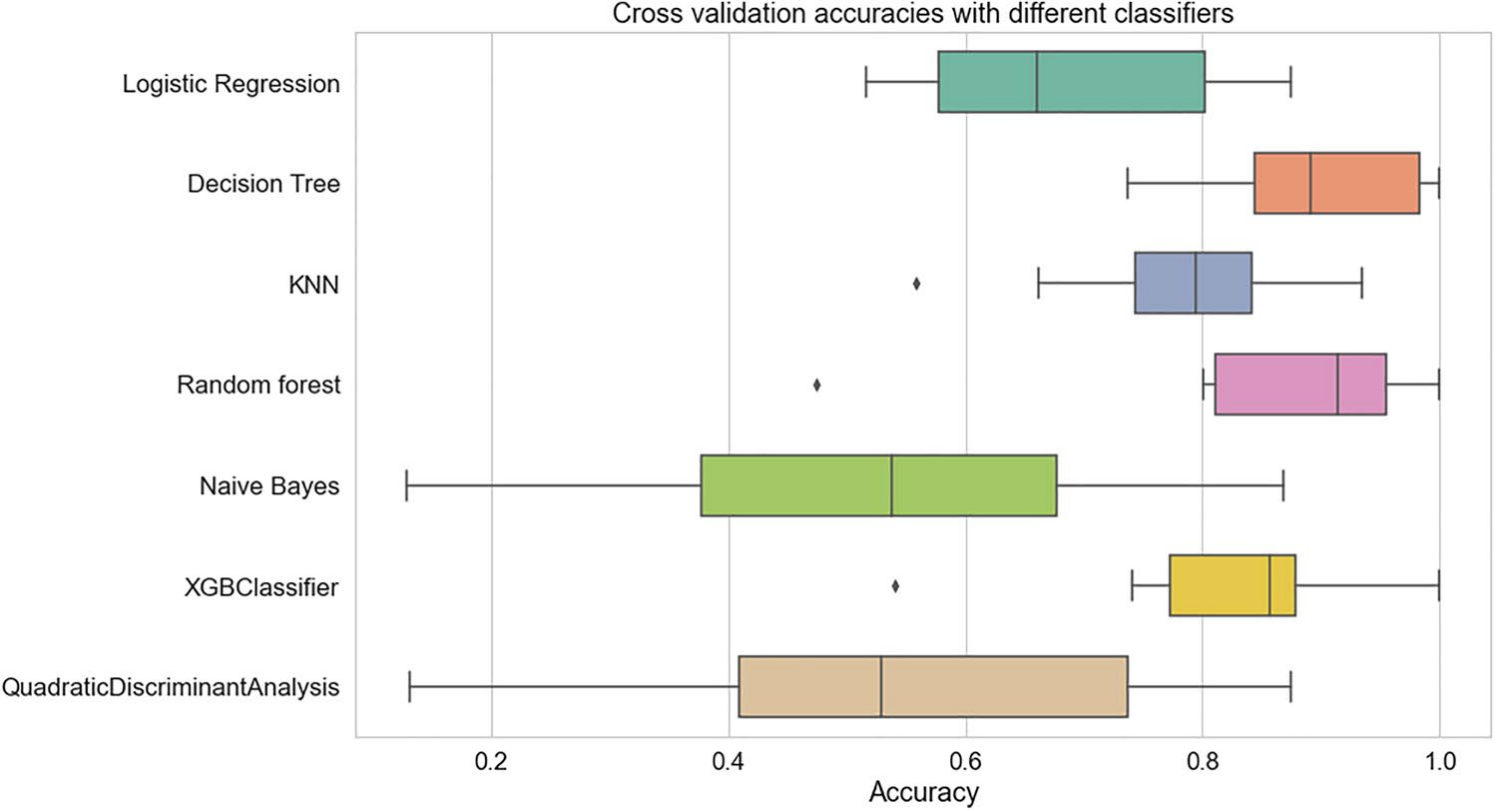
Cross-validation accuracy distribution for different classification models.

To ensure reliability and generalization, fivefold cross-validation was applied. Figure 6 presents the accuracy values obtained across the five folds. Models such as Decision Tree, Random Forest, and XGB exhibited stable performance during both training and testing phases. In contrast, KNN showed larger variance across folds, suggesting sensitivity to data distribution.

### Comparison of ROC(AUC) performance across models

**Fig. 7:**
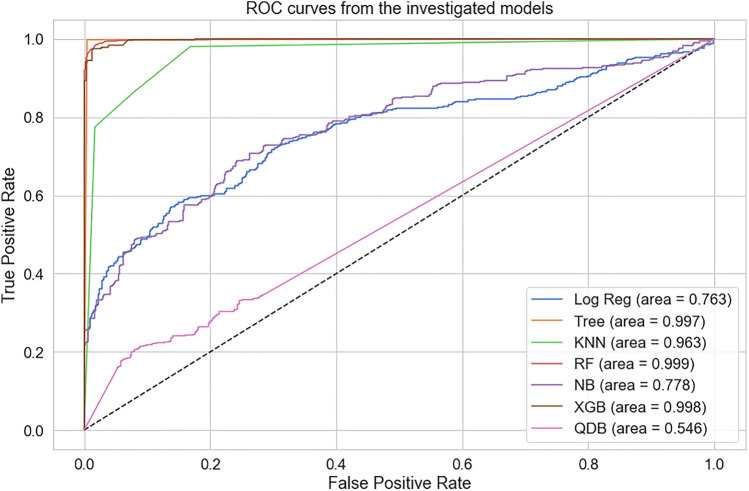
ROC curves and AUC comparison of different machine learning classification models.

Figure 7 compares the ROC curves of different models such as Logistic Regression, Decision Tree, KNN, Random Forest, NB, XGB and QDB. Models with strong ensemble learning capabilities namely RF and XGB achieved the highest AUC values (close to 1.0), reflecting their robustness against misclassification. Logistic Regression, Decision Tree, and KNN showed lower AUC values but remained competitive within their respective model classes.

### Final evaluation of the hybrid random forest–XGBoost model

**Fig. 8:**
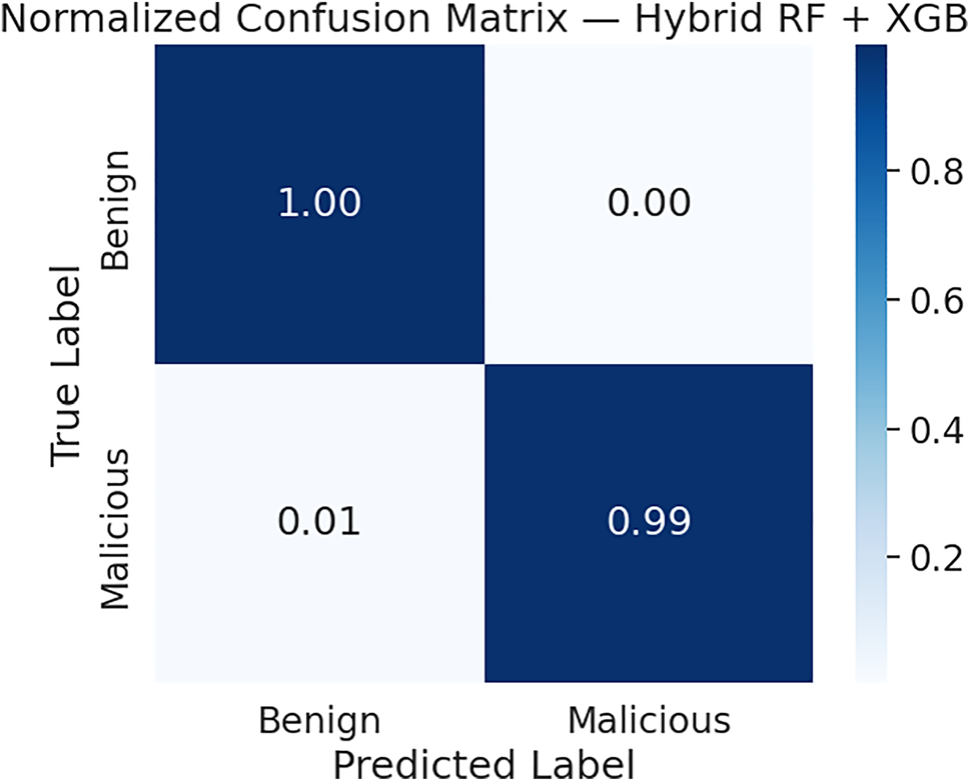
Confusion Matrix for the Hybrid RF + XGB Model.

To provide a comprehensive assessment of the proposed hybrid classifier, an additional evaluation was performed using the optimized Random Forest and XGBoost ensemble. As illustrated in the confusion matrix (Fig. 8), the hybrid model correctly classified nearly all benign traffic and successfully detected approximately 99% of malicious flows, with a very low false-positive rate and a small false-negative rate. These results demonstrate strong performance across all key metrics, including precision, recall, F1-score, and an overall accuracy of approximately 99.3%.

**Fig. 9:**
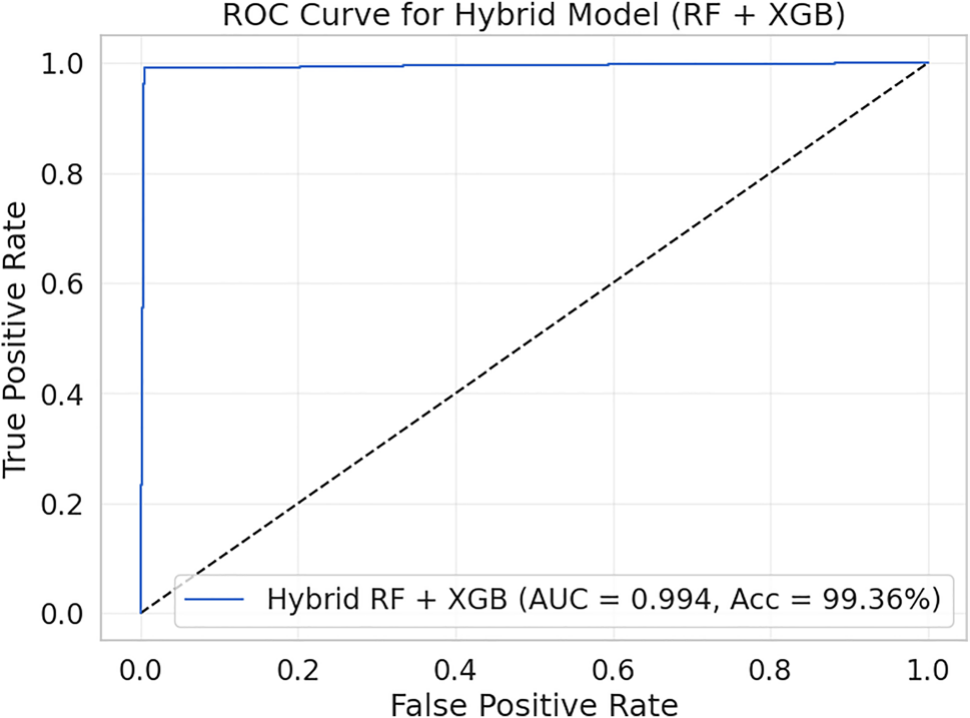
ROC-AUC Curve for the Hybrid RF + XGB Model.

Further, the ROC–AUC curve (Fig. 9) confirms the robustness of the hybrid model. The AUC value approaches 1.0, indicating a steep rise in the True Positive Rate with minimal False Positive Rate across various decision thresholds. This behavior highlights the model’s high sensitivity and specificity and suggests strong discriminative performance on the evaluated dataset.

Overall, the combination of Random Forest and XGBoost leverages the complementary strengths of both algorithms robust feature sampling and gradient boosted decision refinement, resulting in a highly reliable detection engine for SDN environments. These findings reinforce the effectiveness of ensemble based learning when supported by SDN specific feature engineering and validate the suitability of the hybrid model for real time DDoS detection in modern programmable networks.

## Mitigation framework and future integration

The focus of this work is on accurate and early detection of DDoS attacks in Software-Defined Networks using flow- and port-level statistics. While the experiments presented in this paper concentrate on the detection capability of the proposed hybrid model, the same framework can be extended to support automated mitigation in an operational SDN environment.

In a practical deployment, the trained classifier can be integrated with the SDN controller as an additional decision module. As new flows are observed by the controller, the corresponding OpenFlow statistics (e.g., packet counts, byte counts, duration, port bandwidth, and derived features such as APPF and ABPF) can be computed in real time and passed to the classifier. If a flow or group of flows is flagged as malicious, the controller can enact mitigation policies by installing or modifying flow rules on the switches.

A generic mitigation workflow is outlined below:

- **Monitoring:** The controller periodically collects flow and port statistics from OpenFlow switches using OFPFlowStatsRequest and OFPPortStatsRequest messages.
- **Feature Extraction:** For each candidate flow, SDN-specific features are computed, including packet rate, byte rate, number of active flows, and bandwidth indicators.
- **Classification:** The trained hybrid model (Random Forest + XGB Classifier) evaluates the feature vector and assigns a label indicating benign or malicious behavior.
- **Mitigation Decision:** If the flow is classified as malicious, the controller triggers a mitigation policy, such as dropping the flow, rate-limiting it, or blocking the source address.
- **Rule Installation:** Using OFPFlowMod messages, the controller installs high-priority rules on the switch to enforce the selected mitigation action.

Examples of mitigation strategies that can be supported within this framework include:

- **Flow Dropping:** Inserting high-priority rules that match malicious flows and drop packets at the switch, thereby preventing attack traffic from reaching critical resources.
- **Source Blacklisting:** Temporarily blocking traffic from IP addresses identified as persistent attack sources.
- **Rate Limiting:** Applying per-port or per-flow rate limits for suspicious traffic classes to reduce the impact of volumetric attacks while preserving benign flows.

The end-to-end detection-to-mitigation latency in such a system would depend on the monitoring interval, feature extraction overhead, and model inference time. Quantifying this latency and evaluating the impact of different mitigation strategies on network performance are important directions for future work. Implementing the mitigation workflow within the Ryu controller and validating it on larger and more diverse SDN topologies will be considered in the next phase of this research.

## Limitations

Although the proposed hybrid machine learning model demonstrates strong detection performance in the SDN environment, several limitations must be acknowledged to contextualize the findings of this study.

First, the experimental evaluation was conducted on a single-switch SDN topology using Mininet and a Ryu controller. While this setup provides control and reproducibility, it does not fully capture the complexity of large-scale, multi-switch production networks. Traffic dynamics, controller workloads, and flow-table pressures in real deployments may differ significantly from those observed in our emulated environment.

Second, the dataset was generated using synthetic benign and attack traffic within the testbed. Although the use of SDN-specific flow and port statistics provides relevant features, the dataset may not reflect all patterns found in real-world DDoS campaigns, especially multi-vector, low-rate, or stealthy attacks. Generalization to unseen traffic patterns therefore requires further validation using additional datasets or real network traces.

Third, this work focuses solely on the detection of DDoS attacks and does not experimentally evaluate any mitigation actions. Section Mitigation Framework and Future Integration outlines a mitigation framework that can be integrated with the detection model, but the actual implementation, performance overhead, and detection-to-mitigation latency were not measured in this study. These elements are essential for deploying a complete real-time defense system.

Fourth, the model evaluation did not include statistical significance testing such as McNemar’s test or paired t-tests, which would help confirm whether improvements over baseline models are statistically meaningful. Similarly, variance across repeated runs and confidence intervals for all performance metrics were not reported, which may limit the interpretation of robustness under different training conditions.

Finally, the current feature set–while effective–does not incorporate temporal dependencies or long-term behavioral trends that may exist in evolving SDN environments. Advanced models such as LSTMs or graph-based neural networks may be required to capture temporal correlations or topological context not represented in the present dataset.

These limitations highlight several potential avenues for future work, including large-scale evaluation, integration of real-world traffic datasets, implementation of mitigation strategies, robust statistical validation, and the exploration of deep learning architectures tailored for SDN environments.

## Conclusion

Software-Defined Networking (SDN) offers flexibility and centralized control but remains vulnerable to Distributed Denial of Service (DDoS) attacks targeting the controller switch interaction. To address this challenge, we constructed an SDN specific dataset using a Mininet testbed with a Ryu controller and Open vSwitch, enabling the extraction of flow and port-level statistics that accurately capture benign and attack behavior.

Among the evaluated classifiers, the proposed hybrid Random Forest–XGBoost model achieved the highest performance, reaching 99.36% accuracy with strong precision, recall, and F1-scores. The extended evaluation in Section Final Evaluation of the Hybrid Random Forest–XGBoost Model further confirmed its robustness. The confusion matrix reveals a negligible misclassification rate, and the ROC AUC curve approaches 1.0, indicating excellent class separability. Overall, the results demonstrate that the hybrid RF-XGB ensemble is highly effective for detecting DDoS traffic in SDN environments.

Although the study focuses on detection, Section Mitigation Framework and Future Integration outlines how the model can be integrated into an SDN controller to support automated mitigation actions. As discussed in Section Limitations, further work is needed to evaluate scalability, statistical robustness, and real world deployment characteristics.

Future research will investigate deep learning architectures and temporal modeling techniques to broaden threat coverage and enhance adaptation to evolving attack patterns. Overall, the results demonstrate that machine learning, combined with SDN aware feature engineering, offers a practical and effective pathway toward resilient and intelligent SDN security systems.

## Data Availability

The dataset and machine learning code used in this study have been uploaded to a private GitHub repository: https://github.com/ifee57/sdn-ddos-hybrid-ml. Because the manuscript is under peer review, the repository is currently private to prevent premature public release. Access will be granted to the handling editor and reviewers upon request. The repository will be made publicly accessible upon acceptance or publication of the manuscript, in accordance with Scientific Reports’ data transparency policy.
